# NMR Lineshape Analysis of Intrinsically Disordered Protein Interactions

**DOI:** 10.1007/978-1-0716-0524-0_24

**Published:** 2020-01-01

**Authors:** Christopher A. Waudby, John Christodoulou

**Keywords:** Nuclear magnetic resonance, Titrations, IDP, Binding, Kinetics

## Abstract

Interactions of intrinsically disordered proteins are central to their cellular functions, and solution-state NMR spectroscopy provides a powerful tool for characterizing both structural and mechanistic aspects of such interactions. Here we focus on the analysis of IDP interactions using NMR titration measurements. Changes in resonance lineshapes in two-dimensional NMR spectra upon titration with a ligand contain rich information on structural changes in the protein and the thermodynamics and kinetics of the interaction, as well as on the microscopic association mechanism. Here we present protocols for the optimal design of titration experiments, data acquisition, and data analysis by two-dimensional lineshape fitting using the TITAN software package.

## 1 Introduction

Protein interactions are central to their function, whether that be enzymatic, structural, or regulatory. Regulatory proteins or protein domains are particularly prevalent in higher-order organisms, and order-disorder transitions can play important roles in interactions of these proteins [1]. Indeed, intrinsically disordered proteins (IDPs) or intrinsically disordered regions (IDRs)—i.e., polypeptide sequences lacking any stable tertiary structure—have been labelled “interaction specialists” due to their ability to recognize many factors and integrate multiple signals with tuneable affinity and specificity [1, 2]. IDRs are abundant within signalling proteins and oncogenes [3], as well as within viral proteomes [4–6], while other IDPs and IDRs are implicated in a number of severe neuro-degenerative disorders due to their propensity to misfold and aggregate because of their lack of stable structure [7, 8].

The dynamic nature of IDPs and IDRs poses a challenge to most structural biology techniques, but this is an area in which solution-state NMR spectroscopy excels. From the identification of disordered regions and the characterization of their residual secondary and tertiary structure, both in vitro and within living cells [9–12], the study of posttranslational modifications [13, 14], the structural and dynamical characterization of interactions [6, 15–19], to the analysis of phase separation and aggregation [20, 21], NMR spectroscopy has played a key role in the field.

This chapter will focus on the application of NMR lineshape analysis, to characterize interactions of IDPs and IDRs with small molecules or other macromolecules. However, the protocol we provide is equally applicable to interactions of folded protein domains and indeed to interactions of folded domains with IDPs and IDRs.

NMR spectra are sensitive to chemical exchange (i.e., dynamic molecular equilibria) across a wide range of timescales. Chemical exchange between two states can be characterized by the exchange rate, *k*_ex_, which is the sum of the forward and backward reaction rates. For a simple two-state binding reaction in which a protein, P, is observed to form a complex, PL, in the presence of ligand, L:

(1) $$\text{P}\;+\;\text{L}\overset{k_{\text{on}}}{\underset{k_{\text{off}}}{⇌}}\text{PL}$$

The exchange rate is:

(2) $$k_{\text{ex}}=k_{\text{on}}\left[\text{L}\right]+k_{\text{off}}$$

The dissociation constant *K*_d_ = *k*_off_/*k*_on_, and the free ligand concentration $\left[\text{L}\right]=\frac{1}{2}\times \left\{{\left[\text{L}\right]}_{\text{tot}}-{\left[\text{P}\right]}_{\text{tot}}-K_{\text{d}}+\sqrt{{\left({\left[\text{L}\right]}_{\text{tot}}+{\left[\text{P}\right]}_{\text{tot}}+K_{\text{d}}\right)}^{2}-4{\left[\text{P}\right]}_{\text{tot}}{\left[\text{L}\right]}_{\text{tot}}}\right\}$

**Fig. 1:**
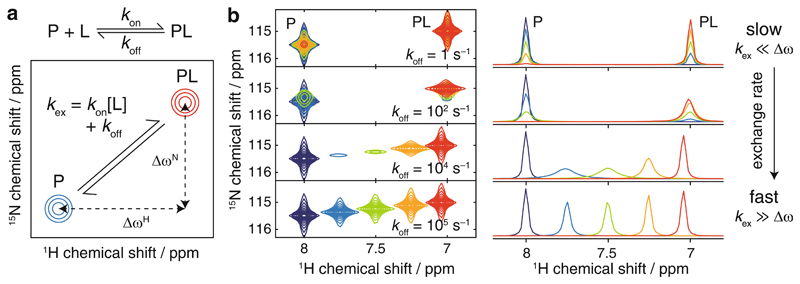
(**a**) Definition of the exchange rate, *k*_ex_, and frequency differences, Δ*ω* ^H^ and Δ*ω* ^N^, for a protein-ligand interaction observed in a two-dimensional heteronuclear correlation experiment. (**b**) ^1^H, ^15^N-HSQC spectra and projected ^1^H 1D cross sections for a simulated protein-ligand interaction (700 MHz, 1 mM protein concentration, *K*_d_ 2 μM, Δ*ω* ^H^ 4400 s^–1^ [1 ppm], Δ*ω* ^N^ 220 s^–1^ [0.5 ppm]) illustrating lineshapes that may arise under various exchange regimes, as indicated. Contour levels are constant across all spectra. Adapted from [40]

The appearance of an NMR resonance depends on the frequency difference between free and bound states, Δ*ω*, relative to the exchange rate (Fig. 1). If exchange is fast (*k*_ex_ ≫ Δ*ω* Am), then a single resonance will be observed with a population-weighted average chemical shift, while if exchange is slow (*k*_ex_ ≪ Δ*ω*), then two signals will be observed at the chemical shifts of the free and bound states, weighted according to the bound population. If the exchange rate is comparable to the frequency difference, a condition termed “intermediate exchange,” then extensive line broadening is observed. As different residues will have different chemical shift differences between their free and bound states, depending on changes in structure or chemical environment of the bound state, a range of fast/intermediate/slow exchange regimes are likely to be sampled across the polypeptide sequence. Note also that the free ligand concentration can only increase as ligand is added, and therefore according to Eq. 2, higher ligand concentrations will be associated with more rapid chemical exchange.

The dependence of the observed NMR spectrum on the *kinetics* of the exchange process as well as the thermodynamics (the bound population) and structure (chemical shift differences and linewidths) is the essential reason why NMR spectroscopy provides a powerful tool to characterize molecular equilibria and binding reactions. The evolution of magnetization in an exchanging system may also be manipulated with rf fields and pulses, which has given rise to a range of methods such as exchange spectroscopy (EXSY) [22–24], CPMG or *R*_1ρ_ relaxation dispersion [25–29], and chemical/dark-state exchange saturation transfer (CEST and DEST) [20, 30, 31], which may be used to characterize dynamic equilibria even within single samples [32]. However, in systems where titrations can be performed, i.e., where the concentration of one or more components can be altered to modulate the equilibrium, NMR titrations using standard 2D correlation experiments provide a powerful approach to the analysis of exchange that is often simpler and more intuitive to analyze than the methods described above.

In the extreme fast exchange limit, which is typically associated with very weak interactions, NMR titration data can be analyzed in terms of chemical shift perturbations alone, which provide a straightforward report of the binding process [33, 34]. In general however, analyses based on a naïve assumption of fast exchange risk the introduction of systematic errors [35]. A better alternative is the use of NMR lineshape analysis (also referred to as “dynamic NMR”) to fit observed spectra to numerical solutions of the Bloch-McConnell or Liouville-von Neumann equations that govern the evolution of magnetization vectors or density operators in the presence of chemical exchange [36, 37].

NMRlineshape analysis was originally developed and applied to one-dimensional NMR spectra [37], but the analysis of proteins and other macromolecules, which contain on the order of 10^3^ ^1^H resonances, clearly requires multidimensional spectroscopy to resolve individual spin systems. Early approaches to lineshape analysis of two-dimensional spectra, which we term “pseudo-two-dimensional lineshape analysis,” were based on the extraction or integration of cross sections through resonances or across rectangular regions, to which traditional one-dimensional lineshape analysis could be applied [38, 39]. However, not only is this approach limited by the need to avoid overlapping resonances—a particularly common problem in crowded spectra of IDPs—but additional uncertainty is introduced by the need to normalize the area under each cross peak, which may by highly broadened by chemical exchange. Lastly, because the pseudo-two-dimensional approach does not accurately consider the evolution of magnetization through the full pulse sequence, we have also shown that this approach can introduce systematic errors by neglecting differential relaxation in the slow-intermediate exchange regime [40] and phase distortions and cross peaks arising from chemical exchange during mixing and chemical shift evolution periods [41].

To resolve these problems, we recently developed a true two-dimensional lineshape analysis approach and introduced a software package, TITAN, to carry out the fitting procedure [40]. The method is based on the use of a “virtual spectrometer” to propagate an initial density operator through the entire experimental pulse sequence [42], to generate a two-dimensional interferogram which can be processed in an identical manner to the experimental data. This approach eliminates the systematic errors described above; obviates the requirement to normalize individual resonances or spectra, provided that sample concentrations and acquisition parameters are fully specified; and can readily fit overlapping resonances, which is of particular value for IDPs. The analysis has since been applied to a range of systems [43–46].

Here, we present a protocol for the acquisition and analysis of NMR titration data to characterize an IDP interaction with a ligand (which may be a small molecule or another macromolecule) using two-dimensional lineshape analysis. We begin with a discussion of the experimental setup and optimal titration protocols and then present a step-by-step description of data analysis using the TITAN software package. While we highlight some points of particular relevance to IDPs, we note that the protocol is also applicable to other macromolecular systems.

## 2 Materials

### 2.1 NMR Spectrometer

NMR titration experiments may be acquired at any magnetic field strength. However, due to the limited chemical shift dispersion associated with IDPs, it is recommended that NMR spectrometers operating with a ^1^H Larmor frequency of 800 MHz (18.8 T) or greater are used for titration measurements. NMR spectrometers equipped with cryogenic probes will also provide greater experimental sensitivity and allow titrations to be completed more rapidly. Automation facilities, if available, may be used to acquire a complete series of pre-prepared titration samples, albeit at the cost of additional protein and ligand.

### 2.2 NMR Tubes

In our experience, regular 5 mm tubes are most convenient for carrying out NMR titrations, requiring a sample volume of 550 μL. The most crucial requirement in performing a titration is that the sample volume is accurately controlled, and for this reason, we do not recommend the use of Shigemi tubes as inserting and removing the plunger results in uncontrollable sample loss. However, if material is limited, then 400 μL may be used in a 5 mm Shigemi tube without inserting the plunger. Alternatively, if a series of independent samples is being prepared (e.g., due to the inability to prepare a high-concentration stock of titrant), then the use of 3 mm tubes, which require a sample volume of 170 μL, may provide a useful reduction in the total amount of sample required (at the cost of increased acquisition times to compensate for the reduction in experimental sensitivity).

### 2.3 NMR Samples

1. The protein to be observed should be prepared with uniform ^15^N labelling, according to standard expression protocols [47–49]. Although resonance assignments are not strictly necessary for the analysis of titration data, they greatly assist the interpretation of the results, and therefore if assignments are not already available, it is recommended to prepare a second sample with ^13^C/^15^N labelling in order to carry out a backbone assignment according to standard methods [50, 51].
2. Where possible, protein samples should be prepared in a buffer with a strong buffering capacity to avoid pH changes during addition of ligand. Be aware of the risk of protein degradation or aggregation, particularly for IDPs: if necessary, EDTA or protease inhibitors should be added, or the protein concentration reduced, in order to maintain sample integrity across the titration series. As only chemical shift changes are required for the analysis of titration data, rather than absolute values of chemical shifts, we recommend that the chemical shift reference DSS [52] is not included during titration experiments as in a number of cases it may interact with the protein being observed, potentially causing systematic errors in the results [53, 54]. Instead, referencing via the lock frequency has in our experience been found to provide acceptable stability.
3. A high-concentration ligand stock (ca. 5–20× the maximum final ligand concentration, as discussed below) should be prepared in an identical buffer to the protein to avoid changes to solution conditions (especially pH) occurring during the titration. Where the molecular weight of the ligand is sufficiently high, co-dialysis may be helpful, particularly if the IDP contains pH-sensitive histidine residues. Optionally, the ligand stock may be prepared in the presence of the ^15^N-labelled protein, to avoid dilution during the titration, but this is not essential as dilution can also be taken into account during the TITAN analysis. If, for solubility reasons, the ligand is dissolved in DMSO, d6-DMSO is preferred to avoid strong background signals from the solvent, and a control experiment should be performed to verify that DMSO, at the maximum concentration used during the titration, does not perturb the spectrum of the protein being observed.

### 2.4 Software

1. NMRPipe [55] is used for the processing and analysis of NMR data and is available from https://www.ibbr.umd.edu/nmrpipe/.
2. TITAN (v. 1.6) software for two-dimensional lineshape analysis is available from https://www.nmr-titan.com [40]. TITAN requires OS X or Linux to run as a stand-alone application, or it can be run in a scripted manner within MATLAB (version R2016b is currently officially supported).

## 3 Data Acquisition

### 3.1 Sample Concentrations

1. Protein and ligand concentrations must be chosen with some care to ensure accurate results can be obtained from the titration measurement. The protein concentration should not be more than an order of magnitude higher than the *K*_d_, and optimal results are obtained when the protein concentration is comparable to the *K*_d_. The final ligand concentration should be sufficient to fully saturate the binding: the greater of at least three times the *K*_d_ or two equivalents relative to the protein concentration. If even an approximate value of the *K*_d_ is unknown (e.g., from measurements on a related or similar system), an initial titration may be required to obtain an estimate, to be followed up with a more detailed characterization using optimized sample concentrations.
2. Approximately 8–12 titration points should be acquired (*see* **Note 1**). The majority of ligand concentrations should be in the range of 0–1.5 equivalents of protein, unless the protein concentration is much less than the *K*_d_, in which case sufficiently high ligand concentrations should be used in order to saturate the binding (up to ca. 5 × *K*_d_). It is often helpful for ligand concentrations to be spaced nonlinearly, e.g., in quadratic increments: 0, 0.1, 0.25, 0.4, 0.6, 0.85, 1.15, 1.5, and 1.9 equivalents.
3. Protein and ligand stock concentrations should be determined as accurately as possible [56]. Centrifuge protein stocks at ca. 16,000 rpm (20,000 × *g*) for 30 min to remove precipitate or aggregates prior to measurement of concentration. This is most readily done via UVabsorbance measurements (although as IDPs often contain few aromatic residues, alternative methods may on occasion need to be employed [57, 58]). Ensure that the stock concentration or dilution thereof has an absorbance that can be measured with high accuracy, i.e., in the range 0.2–0.6 units. If the ligand stock concentration cannot also be measured spectrophotometrically, ensure that a sufficiently large mass of sample (at least 10 mg) is weighed to avoid errors from the limited precision of the analytical balance. Volumetric glassware or calibrated pipettes should be used for liquid handling; graduated cylinders and plasticware such as serological pipettes and falcon tubes do not provide sufficient accuracy.
4. Protein concentrations of at least 10 μM are required (on modern spectrometers equipped with cryogenic probes), although such low concentrations may necessitate acquisition times of several hours for each spectrum. Higher protein concentrations, around 50–100 μM, will generally allow sufficiently high signal-to-noise ratios to be obtained in less than an hour.
5. A ligand stock should be prepared with a concentration ca. 5–20× that of the final desired concentration, to minimize dilution of the sample (*see* **Note 2**).

### 3.2 Experimental Setup

1. The sample temperature has a strong effect on the quality of 2D NMR spectra and should be optimized before carrying out a titration. Due to the line broadening effect of amide hydrogen exchange with the solvent, which is particularly significant for disordered, solvent-exposed residues, the highest-quality ^1^H,^15^N correlation spectra can generally be obtained for IDPs at lower temperatures, e.g., 278–283 K. This contrasts with typical folded proteins, for which higher temperatures result in more rapid rotational diffusion and hence sharper resonances (*see* **Note 3**).
2. Two-dimensional lineshape analysis proceeds by using a “virtual spectrometer” to calculate the evolution of magnetization through the same pulse sequence that was used to acquire the experimental data. It is therefore important to acquire data with a pulse sequence that is implemented within TITAN. At the time of writing, HSQC and HMQC experiments are fully implemented, but due to the more complex spin dynamics involved in magnetization transfer steps, TROSY, sensitivity-enhanced HSQC, and in-phase HSQC experiments are not compatible and therefore are not recommended (*see* **Note 4**).
  Our preferred ^1^H,^15^N correlation experiments are the SOFAST-HMQC [59, 60] and the HSQC (specifically, the FHSQC variant [61]). The SOFAST-HMQC provides excellent experimental sensitivity with short acquisition times and good resolution in *t*_1_ as the amide selective ^1^H refocusing pulse also refocuses the ^3^ *J*_HNHA_ scalar coupling. However, transverse relaxation and chemical exchange broadening are generally more severe in the indirect dimension of HMQC experiments compared to the HSQC, and therefore in some cases, the HSQC may provide more useful or complementary information.
  Whichever experiment is selected, it is important that good solvent suppression and flat baselines are obtained, without the need to apply first-order phase correction in direct or indirect dimensions (with the exception of 90°/180° phase correction for an initial half-dwell delay). Remarkably, this is not always the case for standard library sequences. Bruker format pulse programs are therefore provided with the TITAN download (v. 1.6 onward) for SOFAST-HMQC and HSQC experiments that we have found provide suitable high-quality data. Note that a Reburp rather than an r-Snob refocusing pulse is recommended in the SOFAST-HMQC experiment [60]. Short relaxation delays should be avoided in SOFAST-HMQC experiments used for two-dimensional lineshape analysis (e.g., d1 must be longer than ca. 300 ms), to ensure that resonance intensities are not strongly weighted by longitudinal relaxation, which may vary between free and bound states.
3. Record a preliminary experiment with a wide sweep width, in order to optimize the sweep width and offset. Acquisition times in the direct and indirect dimensions are a balance between obtaining sufficient resolution and avoiding an excessive number of points that may result in slow lineshape fitting. As a rough guide, we suggest acquisition times of 100 ms in the direct dimension and 30–50 ms in the indirect dimension. Folded peaks may also be fitted in TITAN, and this may be used to reduce the sweep width and hence the number of points to be sampled.

### 3.3 Performing the NMR Titration

1. Record a 1D ^1^H spectrum of the unbound protein sample, as a reference, and an appropriate 2D experiment selected and set up as detailed above.
2. Remove the sample from the spectrometer, and add the required amount of ligand stock solution to the sample. Care should be taken while doing this so material is not lost due to adhesion to glass pipettes, Shigemi plungers, etc. Our recommended approach is that the sample should not be removed from the NMR tube, but instead the appropriate volume of ligand stock should be added to the top of the tube, which is recapped and closed with parafilm and then inverted several times to ensure complete mixing. A hand centrifuge can be used to return the sample to the bottom of the tube, and with careful handling, we find that bubbles can generally be avoided (which otherwise would greatly reduce the quality of the spectrum). Alternatively, the ligand may be delivered into the sample and mixed using a narrow metal spatula.
3. Reinsert the sample into the spectrometer, and after allowing time for the temperature to re-equilibrate, lock, shim, and recalibrate the ^1^H 90° pulse length (this is particularly important if the ligand is dissolved in DMSO rather than in the NMR buffer).
4. Record a new pair of 1D and 2D spectra, and repeat from **step 2** until the titration is complete. Note that for analysis in TITAN, it is essential that acquisition parameters such as the spectrum widths or offsets are not changed between experiments. The number of scans and receiver gain may however be varied between points as required to obtain high-quality spectra.
5. Alternatively, if a series of samples have instead been prepared (e.g., because a high-concentration ligand stock could not be prepared), these may be run sequentially in automation mode. As a quality control measure, in this situation, we also recommend acquiring 1D ^1^H spectra for each sample.

### 3.4 Data Analysis

#### 3.4.1 Analysis of 1D ^1^H Spectra

1D ^1^H spectra acquired across the titration series should be inspected for unexpected changes in protein intensity or linewidth, which may indicate aggregation or degradation. If a buffer was used containing non-exchangeable protons (e.g., Tris, HEPES, acetate), their chemical shifts may be used to verify that the sample pH remained constant throughout the titration.

#### 3.4.2 Processing

2D spectra should be processed in NMRPipe, using linear prediction and exponential window functions. The strengths of the window functions should be chosen as a compromise between eliminating truncation artifacts (“sinc wiggles”) and optimizing sensitivity and minimizing overlap between adjacent signals. To accelerate analysis in TITAN, it is helpful to extract only the ^1^H chemical shift range containing signals to be analyzed, e.g., between 7 and 10 ppm (or less in the case of IDPs). No extraction should be applied in the indirect dimension.

All spectra must be processed in an identical manner. To facilitate this, once fid.com and nmrproc.com processing scripts have been prepared for one spectrum, an automated script may be used to process the remaining spectra, adapted from the example below: 
Listing 1.
#!/bin/csh
# automated processing of 2D spectra in experiments 1 to 11
# processed spectra will be named spectrum-1.ft2, spectrum-2.
ft2, ...
foreach spec (1 2 3 4 5 6 7 8 9 10 11)
# conversion from Bruker to nmrPipe format, adapted from fid. com:
bruk2pipe -in ./$spec/ser \
 -bad 0.0 -noaswap -AMX -decim 16 -dspfvs 12 -grpdly -1 \
 -xN 2048 -yN 256 \
 -xT 1024 -yT 128 \
 -xMODE DQD -yMODE States-TPPI \
 -xSW 9615.385 -ySW 1823.985 \
 -xOBS 599.927 -yOBS 60.797 \
 -xCAR 4.611 -yCAR 118.959 \
 -xLAB HN -yLAB 15N \
 -ndim 2 -aq2D States \
 -out ./test.fid -verb -ov
# 2D processing, adapted from nmrproc.com
# with: solvent suppression via SOL filter
# 4 Hz and 8 Hz exponential line broadening
# extraction of 1H dimension from 7--10 ppm
# linear baseline correction in 1H dimension
# linear prediction in indirect dimension nmrPipe -in ./test.fid \
| nmrPipe -fn SOL \
| nmrPipe -fn EM -lb 4.0 -c 0.5 \
| nmrPipe -fn ZF -auto \
| nmrPipe -fn FT -auto \
| nmrPipe -fn PS -p0 152.00 -p1 0.00 -di -verb \
| nmrPipe -fn EXT -x1 7ppm -xn 10ppm -sw \
| nmrPipe -fn BASE -nw 10 -nl 0% 2% 98% 100% \
| nmrPipe -fn TP \
| nmrPipe -fn LP -fb \
| nmrPipe -fn EM -lb 8.0 -c 1.0 \
| nmrPipe -fn ZF -auto \
| nmrPipe -fn FT -auto \
| nmrPipe -fn PS -p0 -90.00 -p1 180.00 -di -verb \
 -ov -out ./spectrum-$spec.ft2
rm ./test.fid
end



#### 3.4.3 Two-Dimensional Lineshape Analysis

**Fig. 2:**
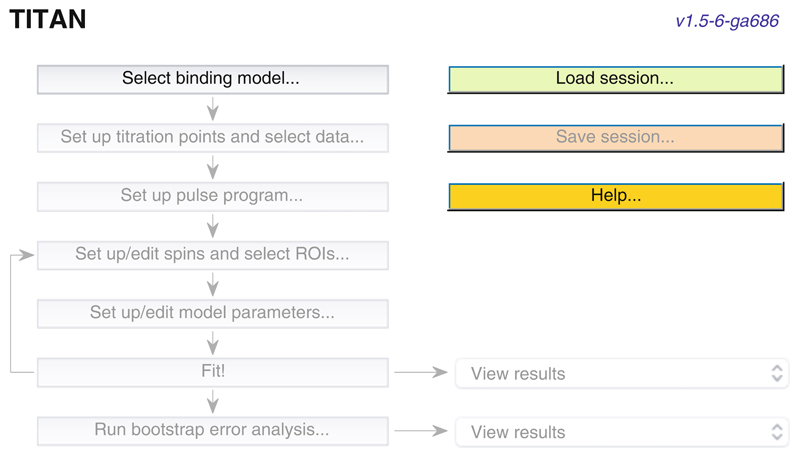
Screenshot of the main TITAN interface. The workflow, indicated by arrows, is progressively enabled as the user proceeds through the analysis

**Fig. 3:**
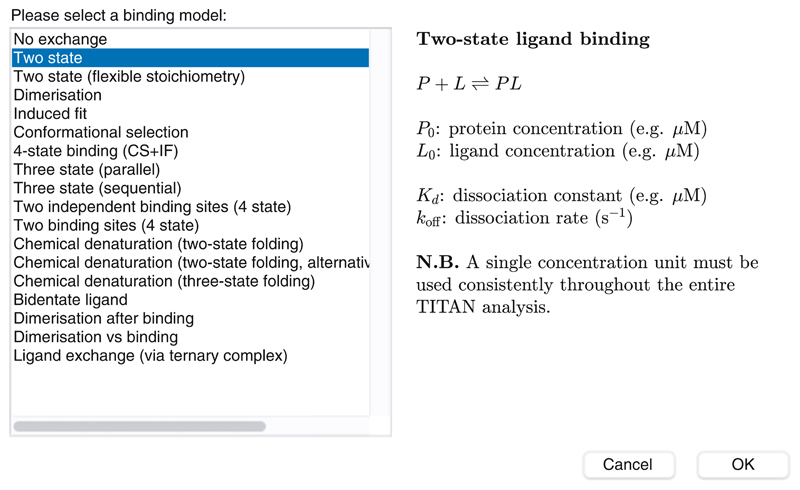
Selection of a binding model. The two-state binding model selected here is suitable for simple protein-ligand interactions, but a variety of additional models are available as shown here and described in Table 1

**Fig. 4:**
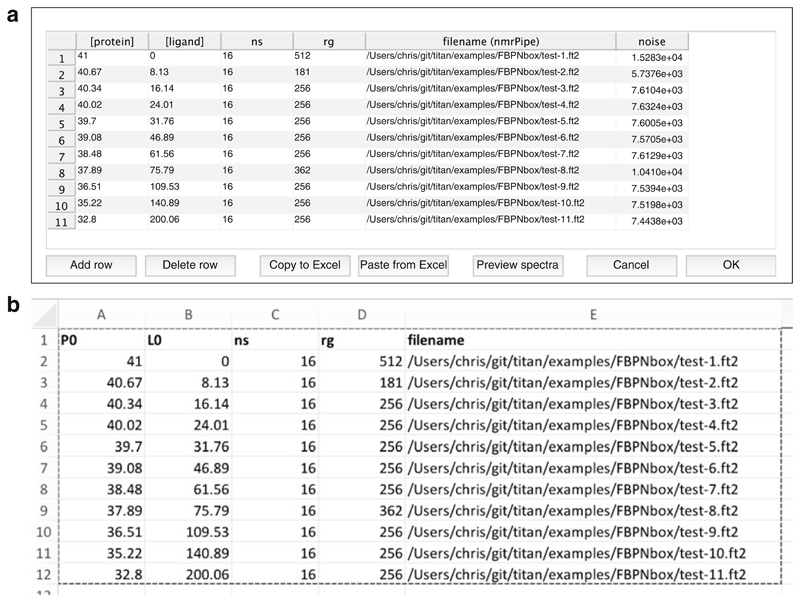
(**a**) Titration setup dialog. Protein and ligand concentrations (as required by the particular binding model selected) must be specified together with the acquisition parameters ns (number of scans) and rg (receiver gain). Spectrum noise levels are required to accurately weight residuals between spectra and are calculated automatically upon importing data. (**b**) For convenience, data for this dialog may be copied and pasted from a suitable Excel spreadsheet

**Fig. 5:**
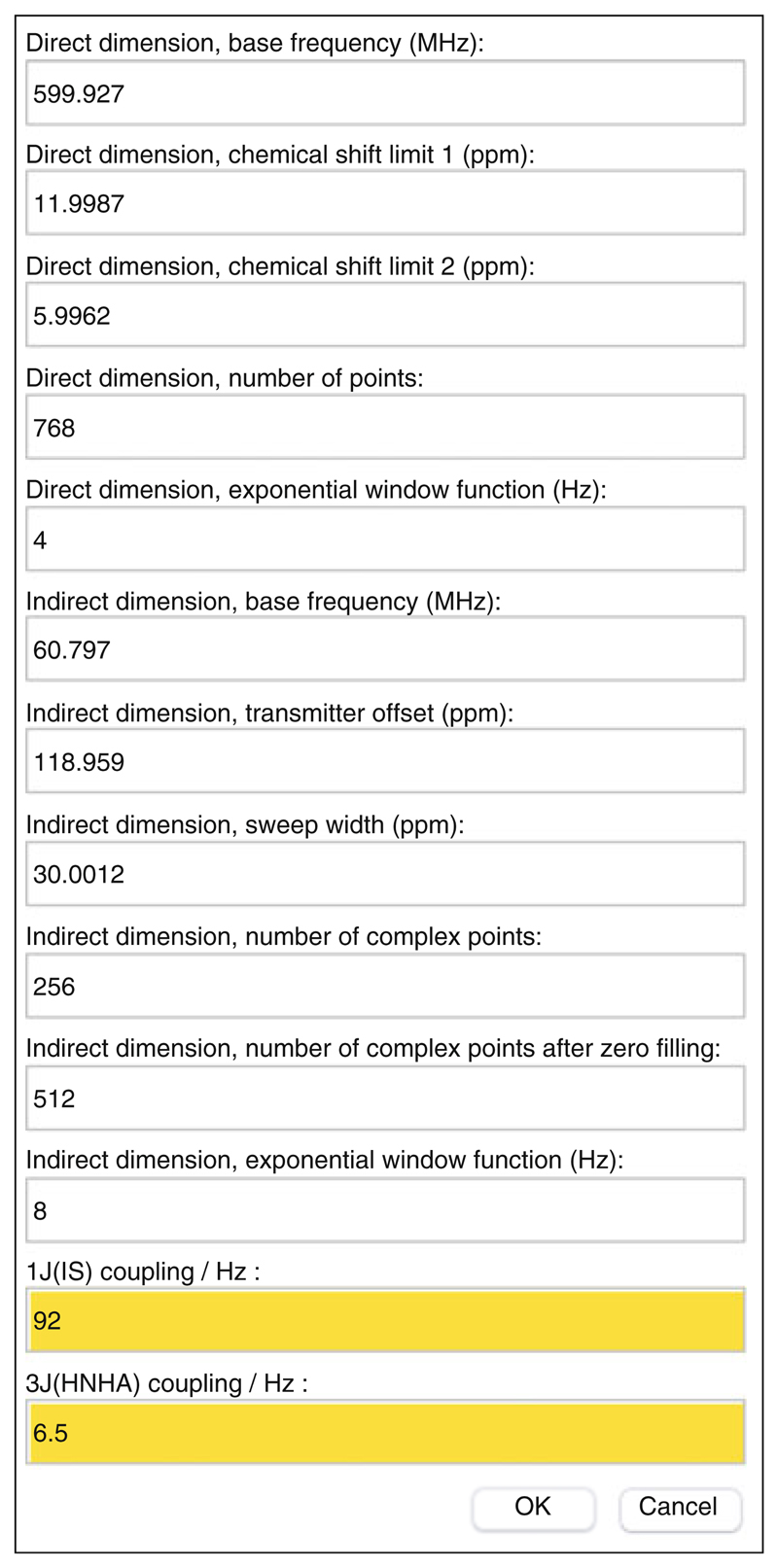
Pulse sequence setup dialog. All fields should be parsed automatically from NMRPipe input files, except those highlighted yellow, for which appropriate values should be set by the user

**Fig. 6:**
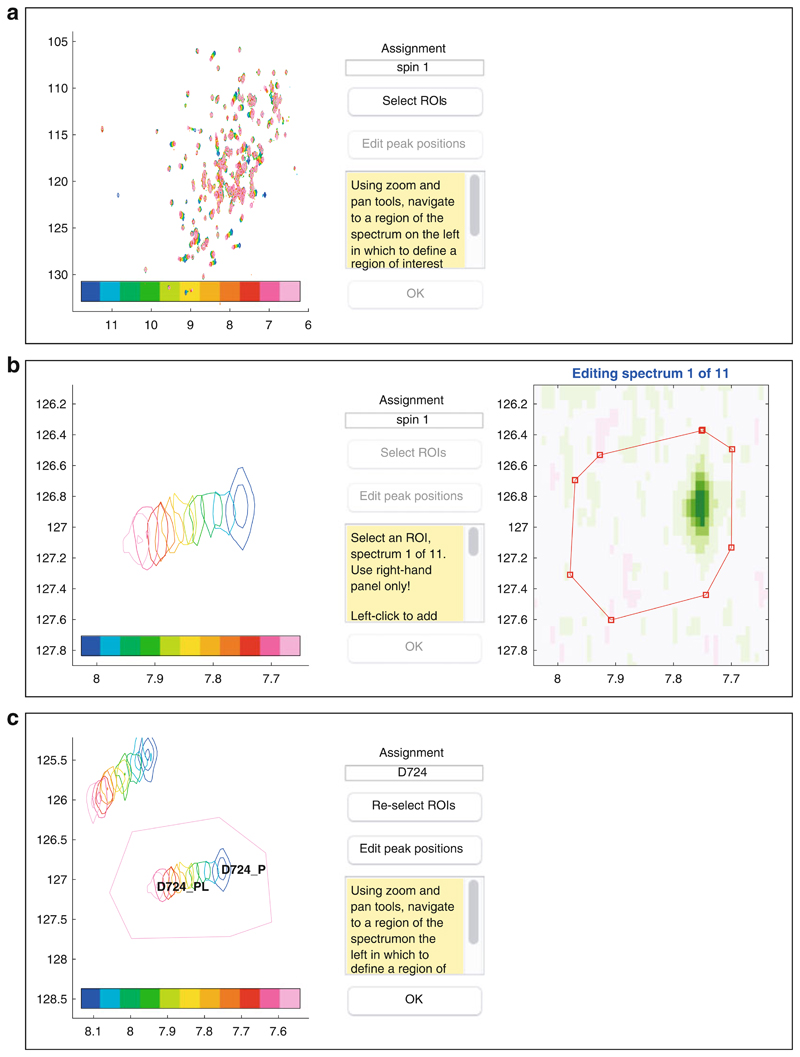
Screenshots of the ROI editor illustrating the setup of a new spin system. (**a**) When first opened, the editor shows an overlay of all spectra in the left-hand panel which may be zoomed and panned using the controls in the toolbar (toolbar not shown). (**b**) ROIs are marked out as a series of points in the right-hand panel. (**c**) Final state of the editor after selecting initial estimates of the free and bound state peak positions

**Fig. 7:**
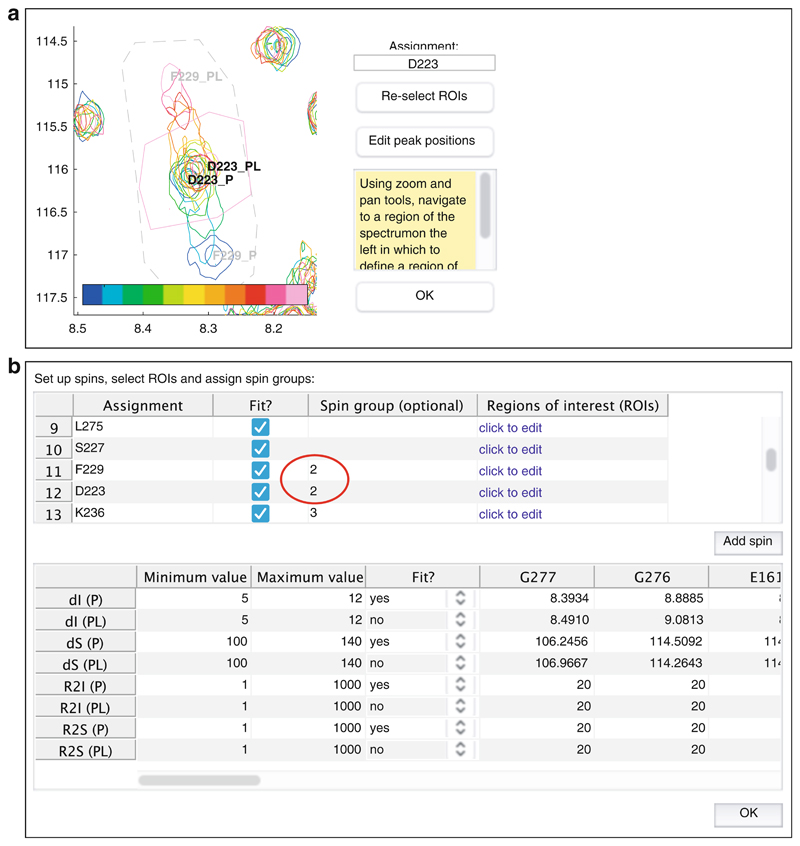
Setting up spin groups for the fitting of overlapping resonances. (**a**) Two residues, D223 and F229, have been defined with overlapping ROIs within the ROI editor. (**b**) The overlapping residues have been associated with a common “spin group” within the spin system editor (red circle). The spin group is an arbitrary text label. ROIs for residues within the same spin group will be merged, and (overlapping) resonances therein will be fitted simultaneously

**Fig. 8:**
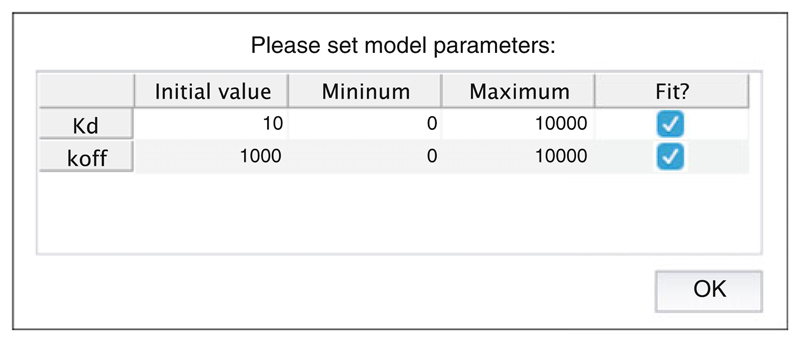
Screenshot of the model parameters editor. Initial values for parameters such as *K*_d_ and *k*_off_ can be specified, together with the allowable parameter range (e.g., which may be constrained on the basis of prior knowledge). Fitting of particular parameters may be activated and deactivated using the checkboxes

**Fig. 9:**
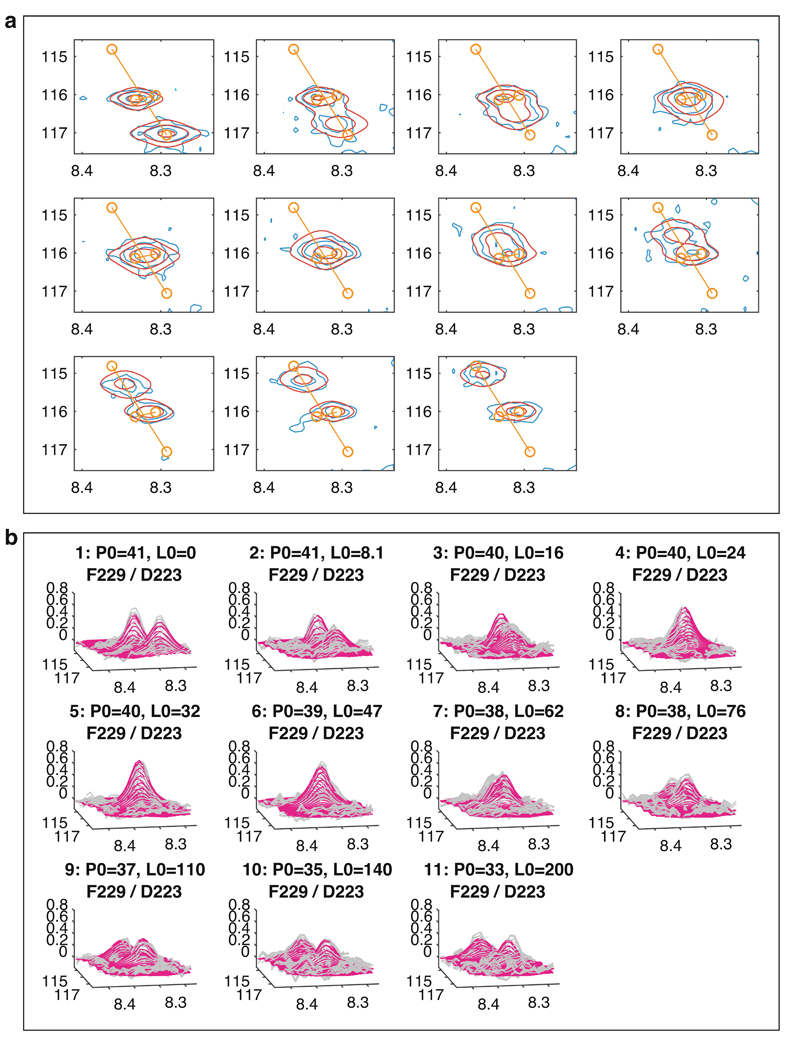
Visualization of fitting results. (**a**) Overlaid contour plots of observed and fitted spectra (blue and red, respectively). (**b**) Three-dimensional views of observed and fitted spectra (gray and magenta, respectively)

**Fig. 10:**
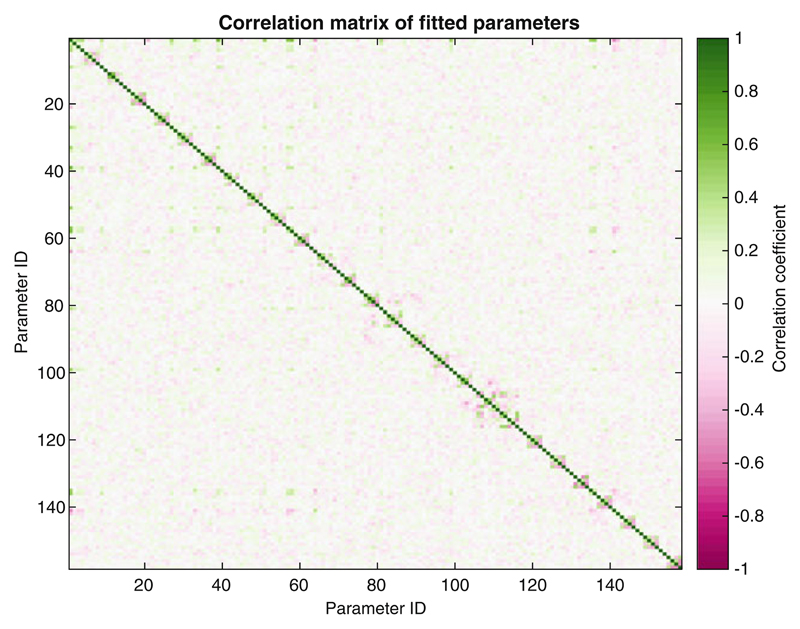
Density plot of the parameter covariance matrix derived from bootstrap error analysis. Parameter IDs are listed within the fitting output and can be explored interactively using the mouse cursor

1. Launch TITAN, either via the pre-compiled binary installation or within MATLAB using the command “TITAN” (having added the TITAN directory to the path as described in the documentation). The main interface is shown in Fig. 2 and provides a directed path through the analysis procedure.
2. “Select binding model...” will launch a dialog to specify the microscopic association mechanism to which experimental data will be fitted (Fig. 3). A number of binding models are available, describing a variety of situations. Some common models are summarized in Table 1.
3. “Set up titration points and select data... ” will open a dialog to import experimental titration data (Fig. 4a) (*see* **Note 5**). For convenience, this data may be copied and pasted from an Excel spreadsheet, the format of which is shown in Fig. 4b. Depending on the binding model selected, protein and ligand concentrations must be specified for each titration point (corrected for sample dilution), as well as the number of scans (ns) and receiver gain (rg) used for each experiment. The experimental data should be in the form of NMRPipe format (.ft2) files generated by the processing steps above. Noise levels will be calculated automatically for each experiment, based on maximum likelihood estimation of a truncated Gaussian distribution, excluding intense regions associated with peaks. These can be manually overwritten if necessary. Note that accurate noise levels are critical for correct weighting of residuals across multiple spectra.
4. Select the pulse sequence used for data acquisition (“Set up pulse program...”). Several pulse sequences are available for analysis within TITAN, of which HSQC and HMQC are the most common experiments. It is important that the pulse sequence is correctly specified as this will strongly affect the results, as chemical exchange can have different effects on line-shapes in the indirect dimension of HSQC and HMQC experiments [40], as well as during coherence transfer periods [41].
  Having selected an experiment type, further acquisition and processing parameters must be specified (Fig. 5). These parameters include the spectrometer frequency, frequency offsets, spectrum widths, the number of points in each dimension (after zero filling and linear prediction, if applied), and the exponential line broadening applied during processing. These parameters should all be correctly parsed from the input NMRPipe format data, except two that must be specified manually: the ^1^ *J*_IS_ heteronuclear scalar coupling between the spins being observed (ca. 92 Hz for amide spin systems) and the value of the ^3^ *J*_HNHA_ scalar coupling that is active during the final acquisition step. For fully protonated amide spin systems, the approximate value of 6.5 Hz has been sufficient for all cases the author has examined to date. However, if the protein being observed is perdeuterated, then the ^3^ *J*_HNHA_ coupling should be set to zero at this point (*see* **Note 6**).
  Some experiments, such as the HSQC, will prompt for further details on delays within the particular pulse sequence employed, which are required to correctly propagate chemical exchange through gradient selection delays and zz filters. If the HSQC sequence provided is used, then the default parameters will be correct.
5. Open the spin system editor (“Set up/edit spins and select ROIs...”). This interface is used to select residues and regions of the experimental spectra that are used for analysis. Spin systems are a key concept within TITAN: each spin system represents a single residue and, for every state specified by the binding model (e.g., free and bound states), carries information on direct and indirect chemical shifts (dI, dS) and line-widths (R2I, R2S) (*see* **Note 7**). Optionally, spin systems can be labelled with an assignment; otherwise a default numbered label is created. For each spectrum, a spin system is associated with a *region of interest* (ROI). This is an area of the spectrum that will be simulated and used for fitting of the associated spin system.
  When the spin system editor is first opened, a new spin will be created automatically and the ROI editor launched (Fig. 6). The left-hand panel displays an overlay of all the experiments in the titration series, and any existing ROIs will also be marked on this panel. Use the toolbar to zoom in to the residue of interest, adjusting the contour levels as necessary, and then press “Select ROIs” to begin marking out ROIs. The first experiment will be displayed as a density plot in the right-hand panel; the color scale may be adjusted using the up/down arrow keys. Left click in this panel to mark out a polygon defining the boundary of the ROI; a right click or the space bar will add a final point and close the boundary. This process is then repeated for the remaining spectra. Distinct ROIs can be defined for each spectrum or alternatively the previous ROI copied using the “c” key (*see* **Note 8** for a discussion of the optimal shapes of ROIs).
  Once ROIs have been selected for all spectra, initial estimates of peak positions are selected for all states in the binding model (e.g., free and bound) by clicking at the appropriate position in the left hand panel. By default, linewidths of all states are set to 20 s^–1^, which generally provides an acceptable starting point for fitting (*see* **Note 7**).
  Where resonances of multiple residues overlap, there is no restriction on ROIs also overlapping. However, these spin systems should then be associated together into a “spin group,” by assigning an arbitrary label (e.g., “group 1,” “group 2”) to the relevant spin systems within the upper panel of the spin system editor dialog (Fig. 7).
6. Having created a series of spin systems and associated ROIs, before closing the spin system editor, select the parameters that should be used for fitting using the lower half of the dialog (Fig. 7b). Two-dimensional lineshape analysis involves the fitting of many parameters, and so it is often helpful to carry out the fitting in two stages: firstly, to fit only the free-state chemical shifts and linewidths using the first (unbound) spectrum only and then to fix these chemical shifts and fit the remaining parameters, together with model parameters such as the dissociation constant *K*_d_ and the dissociation rate, *k*_off_, using the complete dataset.
7. Depending on the binding model selected above, a number of model parameters must be specified, which represent thermodynamic equilibrium and kinetic rate constants, such as *K*_d_ and *k*_off_ values. These can be edited by selecting “Set up/edit model parameters....” If initially only the first spectrum is being used for fitting unbound chemical shifts, as described above, then fitting of model parameters should be turned off in this dialog (Fig. 8).
8. After following the preparatory steps above, the “Fit!” command will now be enabled. Fitting will overwrite parameter values, and so it is recommended to save the session before proceeding. Upon proceeding, a dialog will prompt to select spectra to be used in the fitting process (e.g., the first spectrum only for the initial optimization of unbound peak positions).
  While the fitting process is running, a plot of the chi-square residuals is displayed to show the progress of the optimization algorithm, and on completion a list of the fitted parameters will be displayed in a new window. Parameter labels are of the form “ASSIGNMENT_QUANTITY_MODEL STATE.” Note that the reported error comes from the estimated covariance matrix, and the use of bootstrap resampling methods (below) is recommended for more robust estimates.
9. At this stage, the fitting process may be repeated as indicated in the workflow in the main window (Fig. 2), after adjusting the fixed and free parameters as required.
10. Once fitting is complete, a variety of plots are available to assess the quality of the fit. Overlaid contour plots of observed and fitted spectra are a straightforward way to compare the goodness of fit (Fig. 9a), but deviations in peak intensities – which may be a signature of a more complex binding mechanism—are not always obvious in such plots. Therefore, it is also useful to examine interactive overlays of 3D waterfall plots, which may give better insights into signal-to-noise levels and whether intensities are being fitted accurately (Fig. 9b). All plots may be saved as publication quality vector graphics (eps format) using the toolbar. Lastly, fitted (simulated) spectra may be exported into NMRPipe format for visualization and analysis with other software packages such as CCPN Analysis or Sparky [62, 63].
11. Finally, once the fitting has been completed, the option to run a bootstrap error analysis will be enabled. This will repeat the previous fitting step, with the same starting parameters as used previously, using a series of spectra generated through resampling of residuals from the best-fit spectrum [40]. The user is prompted for the number of bootstrap replicas to generate and fit; at least 50 are recommended to obtain reliable estimates of parameter uncertainties.
  Once complete, a summary report is generated containing the mean and standard error of the fitted parameters. The fit results from individual bootstrap replicas can also be tabulated, but this information is perhaps more usefully displayed in the form of the parameter covariance matrix (Fig. 10).
  Parameters of interest should be inspected for strong correlations that may point to weaknesses or hidden uncertainties in the fitting procedure. For example, for a spin system in fast exchange, it may be difficult to differentiate between a relatively high-affinity interaction with a small chemical shift difference between free and bound states and a lower-affinity interaction with a larger chemical shift difference, particularly if the available titration data did not reach saturation. In this situation, a strong correlation would therefore be expected between the fitted *K*_d_ and the fitted chemical shift of the bound state. In general, however, an analysis of multiple spin systems results in a greatly improved covariance structure. This points to the importance of globally fitting multiple spin systems exhibiting fast, intermediate, and slow chemical exchange or, where this is not possible, to ensuring that sufficient ligand is added to weak binding systems in fast chemical exchange so that they reach saturation and an accurate estimate of the bound state chemical shift can be determined.
12. The point at which a fit result is regarded as “acceptable” is ultimately subjective. A typical ROI contains ~500 points, resulting in ~10^5^ observations fitted to ~10^2^ parameters (given 20 ROIs fitted across 10 spectra), and given the complexity of this simulation and fitting process, it is difficult to devise a simple and robust measure of the goodness of fit. Instead, we recommend that the user consider the following criteria in reaching a decision:
  (a) Fitted spectra should accurately reproduce the observed experimental data. This should be assessed using both contour and 3D plots, as discussed above (Fig. 9).
  (b) Selected ROIs should cover the full range of chemical shift differences and chemical exchange regimes observed.
  (c) Sufficient ROIs should have been selected such that the inclusion of additional ROIs does not alter the fitting result (*see* **Note 9**).
  (d) Fitted parameters should have physically reasonable values and not reached their minimum or maximum limits. Where extreme values occur, this indicates that a parameter is not being effectively constrained by the experimental data, and its value should be interpreted with caution.
  (e) If a complex binding model is being fitted, the user should verify that a comparable quality of fit cannot be obtained using a simpler model (i.e., the principle of parsimony).

## 4 Notes

**Fig. 11:**
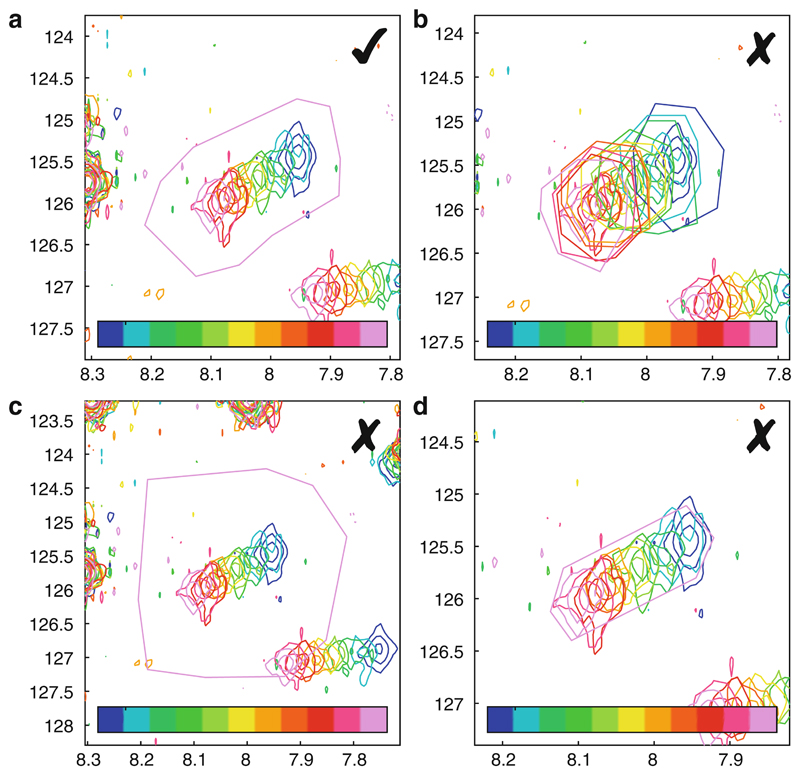
Optimal selection of ROIs. (**a**) Recommended setup: ROIs extend approximately two to three linewidths from the center of resonances and contain the entire region of the spectrum within which resonances are observed across the titration. Note that ROIs are identical for all spectra; hence only a single boundary can be observed. (**b**) Not recommended: individual ROIs do not encircle the entire region within which resonances are observed across the titration. (**c**) Not recommended: too large a selection, resulting in slow fitting, increased noise, and overlap with an adjacent residue. (**d**) Not recommended: too tight a selection, limiting accuracy when fitting linewidths

**Fig. 12:**
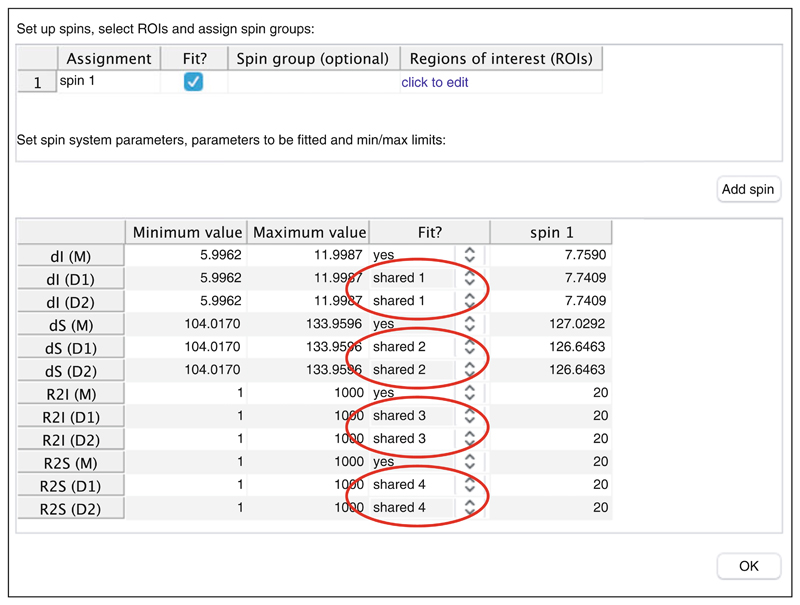
Setting up shared parameters within the spin system editor. The example shown here is a symmetric dimer, defined by linking all properties of the asymmetric dimer states D1 and D2. For each of the D1 and D2 states, the direct and indirect chemical shifts, dI and dS, and the direct and indirect linewidths, R2I and R2S, are assigned to the global parameters “shared 1” to “shared 4” as indicated

1. These guidelines are based on a simple 1:1 association reaction, P + L ⇌ PL. The identification and analysis of more complex binding mechanisms may benefit from a greater number of points or, for example, varying the protein concentration in addition to that of the ligand.
2. If a high-concentration ligand stock cannot be prepared (e.g., due to limited solubility or aggregation), then a lower-concentration ligand stock may be prepared in the presence of the protein, to avoid sensitivity loss due to dilution. Alternatively, a series of individual titration samples should be prepared, either directly (using 3 mm NMR tubes for efficiency) or from serial dilutions of two samples prepared without ligand and with the maximum ligand concentration required. However, we note that in all these cases the total amount of ^15^N-labelled protein that is required is increased.
3. Experiments based on direct ^13^C detection, e.g., CON and CACO, have been developed that provide well-resolved resonances for disordered states under physiological conditions [64, 65], albeit with decreased sensitivity, and titrations have indeed been carried out using such methods [66]. However, these experiments are not currently implemented for two-dimensional lineshape analysis within TITAN and therefore will not be discussed further here.
4. To the extent that relaxation or chemical exchange during magnetization transfer steps can be neglected, other experiments such as transverse relaxation-optimized spectroscopy (TROSY) and sensitivity-enhanced HSQCs may still be analyzed as if acquired with a regular HSQC pulse sequence, but the user should be aware of the additional assumptions involved in such an analysis.
5. For illustrative purposes, data shown in this article use the FBPNbox example provided in the TITAN download.
6. Perdeuteration of proteins (the substitution of all non-labile protons for deuterons, by expression in D_2_O and d7-glucose) is most commonly associated with high molecular weight and slowly tumbling systems, of approximately 30 kDa and above, and is usually applied in combination with TROSY experiments. IDPs and IDRs experience rapid rotational diffusion and therefore do not usually benefit from the application of TROSY experiments. Nevertheless, it has been reported that perdeuteration can significantly improve the quality of IDP spectra, by eliminating the ^3^ *J*_HNHA_ scalar coupling that otherwise increases ^1^H linewidths by 6–12 Hz [67].
7. Linewidths in TITAN are denoted R2I and R2S, in the direct and indirect dimension, respectively. However, these values do not represent exact relaxation rates, but instead reflect approximate combinations of in-phase and anti-phase relaxation rates, as well as deviations from the constant ^3^ *J*_HNHA_ scalar coupling imposed on all residues (defined in the pulse program setup, Fig. 5). Therefore, in practical terms, it is not particularly helpful to set initial values for these parameters based on experimental relaxation measurements, and we recommend that caution should be exercised in any detailed interpretation of fitted values.
8. Our recommendations for the optimal shape and selection of ROIs are illustrated in Fig. 11. ROIs should extend approximately two to three linewidths from the center of resonances, in order that linewidths may be accurately determined (Fig. 11a). We also recommend that all ROIs for a given residue should cover the full region of the spectrum in which its resonances are observed (Fig. 11b), because the *absence* of resonances in such empty regions may in fact represent additional experimental restraints. However, overly large ROIs should be avoided (Fig. 11c), as these will only result in increased noise, slower fitting calculations, and potentially inadvertent overlaps with adjacent resonances. Conversely, tightly cropped ROIs (Fig. 11d) will reduce the accuracy of fitted linewidths and should be avoided.
9. As fitting within TITAN overwrites previous results and redefines the starting point for subsequent fits, there is a risk of becoming trapped in a local minimum when appending new spin systems and ROIs to an existing fit. We therefore recommend saving TITAN sessions before performing fits and reloading these sessions before defining and fitting additional spin systems.
10. Dimers are represented within TITAN using two separate spin states, i.e., the equilibrium 2M ⇌ D is represented internally as 2M ⇌ D_1_ + D_2_. This allows for the possibility that the dimer might be asymmetric [68]. To perform calculations for a symmetric dimer, the chemical shifts and linewidths of the states D_1_ and D_2_ should be linked. This can be done by associating the parameters to a number of available shared variables using the lower panel of the spin system editor (Fig. 12).

## Figures and Tables

**Table 1:** A summary of key binding models implemented within TITAN

Model	Schematic	Fitting parameters	Comments
No exchange	N/A	N/A	Suitable for fitting linewidths and peak positions in single spectra
Two state	$\text{P}+\text{L}\overset{k_{\text{on}}}{\underset{k_{\text{off}}}{⇌}}\text{PL}$	*K*_d_ = *k*_off_/*k*_on_ *k*_off_	Default model for a simple binding reaction. “P” represents the observed protein and “L” the ligand
Two state (flexible stoichiometry)	$\text{P}+n\text{L}\overset{k_{\text{on}}}{\underset{k_{off}}{⇌}}\text{PL}$	*K*_d_ = *k*_off_/*k*_on_ *k*_off_	*n* is a parameter that can be fitted to allow for uncertainty in the ligand stock concentration
Induced fit	$\text{P}\;+\;\text{L}\overset{k_{\text{on}}}{\underset{k_{\text{off}}}{⇌}}{\text{PL}}_{\text{open}}\overset{k_{\text{open}}}{\underset{k_{\text{close}}}{⇌}}{\text{PL}}_{\text{closed}}$	*K*_d_ = *k*_off_/*k*_on_ *k*_off_, *k*_open_, *k*_close_	In the context of IDPs, this mechanism may also describe folding upon binding
Conformational selection	${\text{P}}_{\text{open}}\;+\;\text{L}\overset{k_{\text{on}}}{\underset{k_{\text{off}}}{⇌}}{\text{PL}}_{\text{open}}\overset{k_{\text{open}}}{\underset{k_{\text{close}}}{⇌}}{\text{PL}}_{\text{closed}}+\text{L}$	*K*_d_ = *k*_off_/*k*_on_ *k*_off_, *k*_open_, *k*_close_	In the context of IDPs, this mechanism may also describe binding upon folding
Four-state exchange	$\text{A}+\text{L}\overset{k_{\text{on},\text{A}}}{\underset{k_{\text{off},\text{A}}}{⇌}}\text{AL}\overset{k_{\text{BA}}}{\underset{k_{\text{AB}}}{⇌}}\overset{k_{\text{BA}\prime }}{\underset{k_{\text{AB}\prime }}{⇌}}\text{B}\;+\;\text{L}\overset{k_{\text{on},\text{B}}}{\underset{k_{\text{off},\text{B}}}{⇌}}\text{BL}$	*K*_d,app_ *k*_off,A_, *k*_off,B_ *K*_AB_ = *k*_AB_/*k*_BA_ *K*_AB′_ = *k*_AB′_/*k*_BA′_ *k*_ex_ = *k*_AB_ + *k*_BA_ *k*_ex′_= *k*_AB′_ + *k*_BA′_	Binding via induced fit and conformational selection, for a protein with two states, “A” and “B”. Also applicable to coupled folding and binding. Ligand affinity is specified as *K*_d,app_, the apparent dissociation constant for the equilibrium: (A + B) + L ⇌ (AL + BL)
Dimerization	$2\text{M}\overset{k_{\text{on}}}{\underset{k_{\text{off}}}{⇌}}\text{D}$	*K*_d_ = *k*_off_/*k*_on_ *k*_off_	“M” represents the monomer and “D” the dimer. Dimers may be symmetric or asymmetric (*see* **Note 10**)
Two binding sites	$\text{P}+2\text{L}\overset{k_{\text{on},1}}{\underset{k_{\text{off},1}}{⇌}}{\text{B}}_{1}+\text{L}\overset{k_{\text{off},2}}{\underset{k_{\text{on},2}}{⇌}}\overset{k_{\text{off},3}}{\underset{k_{\text{on},3}}{⇌}}{\text{B}}_{2}\;+\;\text{L}\overset{k_{\text{on},4}}{\underset{k_{\text{off},4}}{⇌}}{\text{B}}_{12}$	*K*_d,1_ = *k*_off,1_/*k*_on,1_ *K*_d,2_ = *k*_off,2_/*k*_on,3_ *K*_d,3_ = *k*_off,3_/*k*_on,3_ *k*_off,1_, *k*_off,2_, *k*_off,3_, *k*_off,4_	Two ligand binding sites, labelled “B_1_” and “B_2_”, with positive or negative cooperativity leading toward the doubly bound state “B_12_”. Closure of the thermodynamic cycle determines *K*_d,4_ = *K*_d,1_ *K*_d,3_/*K*_d,2_
